# Vibrating Mesh Nebulisation of Pro-Antimicrobial Peptides for Use in Cystic Fibrosis

**DOI:** 10.3390/pharmaceutics11050239

**Published:** 2019-05-17

**Authors:** Éanna Forde, Graeme Kelly, Louise Sweeney, Deirdre Fitzgerald-Hughes, Ronan MacLoughlin, Marc Devocelle

**Affiliations:** 1 Department of Chemistry, Royal College of Surgeons in Ireland (RCSI), 123 St. Stephen’s Green, Dublin 2, Ireland; graemekelly@rcsi.ie (G.K.); mdevocelle@rcsi.ie (M.D.); 2 Department of Clinical Microbiology, Royal College of Surgeons in Ireland, RCSI Education & Research Centre, Beaumont Hospital, Beaumont, Dublin 9, Ireland; dfitzgeraldhughes@rcsi.ie; 3 Aerogen Ltd., Galway H91 HE94, Ireland; LSweeney@aerogen.com (L.S.); RMacLoughlin@aerogen.com (R.M.); 4 School of Pharmacy, Royal College of Surgeons in Ireland, 123 St. Stephen’s Green, Dublin 2, Ireland; 5 School of Pharmacy and Pharmaceutical Sciences, Trinity College Dublin, Dublin 2, Ireland

**Keywords:** aerosol, antibiotics, antimicrobial peptides, cystic fibrosis, inhalation, peptide drugs, pulmonary drug delivery, prodrugs, vibrating mesh nebuliser

## Abstract

Background: There has been considerable interest in the use of antimicrobial peptides (AMPs) as antimicrobial therapeutics in many conditions including cystic fibrosis (CF). The aim of this study is to determine if the prodrugs of AMPs (pro-AMPs) can be delivered to the lung by a vibrating mesh nebuliser (VMN) and whether the pro-AMP modification has any effect on delivery. Methods: Physical characteristics of the peptides (AMP and pro-AMP) and antimicrobial activity were compared before and after nebulisation. Droplet size distribution was determined by laser diffraction and cascade impaction. Delivery to a model lung was determined in models of spontaneously-breathing and mechanically-ventilated patients. Results: The physical characteristics and antimicrobial activities were unchanged after nebulisation. Mean droplet size diameters were below 5 μm in both determinations, with the fine particle fraction approximately 67% for both peptides. Approximately 25% of the nominal dose was delivered in the spontaneously-breathing model for both peptides, with higher deliveries observed in the mechanically-ventilated model. Delivery times were approximately 170 s per mL for both peptides and the residual volume in the nebuliser was below 10% in nearly all cases. Conclusions: These results demonstrate that the delivery of (pro-)AMPs to the lung using a VMN is feasible and that the prodrug modification is not detrimental. They support the further development of pro-AMPs as therapeutics in CF.

## 1. Introduction

Antimicrobial peptides (AMPs) are short, cationic, amphipathic, peptides that play a crucial role in the innate immune system of all multi-cellular organisms. Their broad-spectrum activity against both Gram-positive and -negative bacteria has generated significant interest for years in their potential use as antimicrobial agents [1]. The antimicrobial activity of AMPs is based on, but not solely restricted to, their interaction with the bacterial cell membrane and has a lower propensity for resistance to develop compared to classical antibiotics [2]. The latter is an attractive attribute in the context of increasing concerns over the emergence of drug resistance in bacteria such as *Pseudomonas aeruginosa* with the chronic use of antibiotics in conditions such as CF [3]. While promising, issues remain over the selectivity of AMPs for bacterial membranes over mammalian ones (and associated host toxicity) and have previously led, among other approaches, to the development of prodrugs of AMPs, targeted for activation by the enzyme neutrophil elastase (NE). This approach, based on a net charge-reducing, anionic pro-moiety, was designed to limit the potentially toxic effects of the AMP to the endobronchial space, where NE is abundant and the *P. aeruginosa* infection is localised in CF. It was successfully implemented in both in vitro and in vivo experiments [4,5].

These (pro)-peptides are designed for local delivery to the lung by inhalation in CF patients in the same manner as antibiotics such as tobramycin, aztreonam lysine and the cyclic polypeptide colistin [6]. This route has been shown to lead to sputum levels of antibiotics that are over 1000 times higher than those produced via intravenous delivery [7]. This is an attractive option in general for AMPs, considering their unfavourable pharmacokinetics and high cost of manufacture [2]; peptides generally have poor oral bioavailability due to their high molecular weight and susceptibility to extreme pH levels/enzymatic degradation [8]. Specifically for these pro-AMPs, inhaled delivery allows direct access to the high levels of the activating enzyme NE on the respiratory surface, the concentrations of which can approach over 100 μM in vivo in CF [9], although varying greatly from patient to patient [10]. This approach also has the potential to limit residual host-toxic effects of the activated peptides to the endobronchial space. However, the ability to deliver these peptides by inhalation must be demonstrated and there are several considerations in terms of the nebulisation system.

The physicochemical properties of the inhaled drug, as well as the formulation, can affect its deposition in the lung. For solutions, droplet size is crucial, a mass median aerodynamic diameter (MMAD) (the diameter at which 50% of the particles by mass are larger and 50% are smaller) of between 1 and 5 μm is required for lower airway deposition. At size ranges lower than this, the particle is likely to be exhaled, and at higher size ranges, it will physically impact the throat and only lower amounts will reach the lung [11]. More specifically, a particle diameter below 3 μm is generally used to target the alveoli, the thinness of the epithelium there making it an attractive target for systemically-active peptides [12]; however, for local delivery, this is not desirable, as in the present case of pro-AMPs.

The large number of drug treatments required and extensive inhalation times in CF mean that patients have a high treatment burden [6]. Ideally, the delivery method would be fast, and compatibility with the peptides is an absolute necessity. Conventional jet nebulisation can exert high shear on compounds (99% of droplets are recycled into the drug reservoir), which has been shown to lead to the denaturation of proteins [12]. Ultrasonic nebulisers, although preferable to jet nebulisers in terms of speed, can cause significant heat generation, presenting problems for heat labile biomolecules such as recombinant human deoxyribonuclease (rhDNase) [13]. Both jet and ultrasonic nebulisers have been shown to denature the protein lactate dehydrogenase [14]. Another option, and one that is preferable for patients based on portability and speed, are vibrating mesh nebulisers (VMNs) [15]. These move the drug solution through a perforated mesh, creating homogenously sized particles for inhalation, allowing for more consistent dosing [16]. They do not rely on the recirculation of droplets (unlike jet and ultrasonic nebulisers) and are reported to generate less heat and shear stress [17]. Like ultrasonic nebulisers, they are fast; it has previously been shown that rhDNase delivery time can be reduced by using a VMN instead of a jet nebuliser [18] and halved in the case of tobramycin [19]. The residual volume of the drug solution in VMNs is also small (<0.1 mL), an important advantage for costly therapeutics such as peptides [20].

Despite their benefits over-jet and ultrasonic nebulisers, compatibility with VMNs is also not guaranteed. There are still thermal and interfacial stresses involved that may interfere [21]. Aggregation and/or loss of activity have been shown to occur with lactate dehydrogenase and IgG1 in VMNs, the latter of which is typically thermostable [17]. Conversely, some drugs can survive harsher nebulisation conditions, colistin has been shown to maintain comparable MICs after both jet and ultrasonic nebulisation, for example, Reference [22]. Further, there are reports of VMN facilitating greater delivered doses to the lung across a variety of interventions [23,24,25]. All things considered, VMNs represent an attractive option.

The present study was performed to evaluate whether it is possible to deliver pro-AMPs via VMN. The droplet size of the aerosol spray and delivery to model lungs were investigated, as were the physical characteristics and activity of the peptide post-nebulisation. A comparison was also made between the active AMP sequence (i.e., representative of that cleaved by NE) and the pro-AMP to determine if the anionic pro-moiety has any impact on nebulisation. There is currently a very limited discussion in the literature on the nebulisation of AMPs and this study represents the first, to our knowledge, investigating pro-AMPs. The results represent an important step in the future development of this class of antimicrobial therapeutics for CF and other applications.

## 2. Materials and Methods

### 2.1. Materials

The Fmoc-protected amino acids and the Rink Amide MBHA resin were obtained from Merck Novabiochem (Nottingham, UK). HATU was purchased from ChemPep Inc. (Wellington, FL, USA). NMP was sourced from BioSciences (Dublin, Ireland). All other reagents and solvents were supplied by Merck Sigma-Aldrich (Dublin, Ireland).

The laboratory strain PAO1 (American Type Culture Collection, Rockville, MD, USA) was used as a reference. *P. aeruginosa* clinical isolates from CF patients (PABH01-03) were obtained from the Microbiological Diagnostic Laboratory of Beaumont Hospital, Dublin, Ireland. Isolate identity was confirmed by a combination of the BBL^TM^ DrySlide^TM^ oxidase test (Becton, Dickinson and Company, Franklin Lakes, NJ, USA), the C-390 Diatab^TM^ disk test (Rosco Diagnostics, Germany), and Matrix Assisted Laser Desorption Ionisation—Time of Flight (MALDI-TOF) mass spectrometry (Microflex LT, Bruker, Germany).

### 2.2. Peptide Synthesis

Two peptides modified by C-terminal amidation were investigated; AAG-D-WMR^3,6-leu^ (AAGwglrrllkygkrs) and its pro-AMP, pro-D-WMR^3,6-leu^ (Ac-EEEEAAAGwglrrllkygkrs). These peptides are referred to hereafter as AAG-WMR and pro-WMR, respectively.

The parent WMR sequence was assembled by automated Solid Phase Peptide Synthesis on a 433A synthesiser (Applied Biosystems, Warrington, UK) from Fmoc-protected D-amino acids with HATU/DIEA coupling chemistry from a Rink Amide MBHA resin. For the pro-AMP, elongation with the AAAG linker, tetraglutamate motif and N-terminal acetylation were carried out manually, with L-alanine and L-glutamic acid. These peptides were used for experiments carried out with 1 mL of the sample. Peptides used in experiments carried out with 2 mL of the sample were produced on a 100 mg scale by Almac (Craigavon, UK).

### 2.3. HPLC Analysis

Chromatographic analysis and purification were performed on a Galaxy HPLC system (Varian, Walnut Creek, CA, USA) and a BioCAD SPRINT Perfusion Chromatography Workstation (PerSeptive Biosystems, Warrington, UK), respectively, using Gemini (5 µm, 110Å, C18) analytical (flow rate 1 mL/min) and semi-preparative (flow rate 5 mL/min) columns (Phenomenex, Macclesfield, UK). The mobile phase consisted in water with 0.01% TFA (buffer A) and acetonitrile with 0.01% TFA (buffer B) and had the following composition: 95% buffer A and 5% buffer B for 5 min; linear gradient from 95% buffer A and 5% buffer B to 35% buffer A and 65% buffer B over 30 min; 35% buffer A and 65% buffer B for 10 min. UV dual-wavelength detection was performed at 214 and 280 nm for the Biocad Sprint, while the Varian Galaxy was equipped with a Diode Array Detector (PDA) operating from 190 nm to 950 nm (chromatograms were recorded at 214 nm). Purified peptides were finally characterised by analytical HPLC and MALDI-TOF MS using the α-cyano-4-hydroxy-cinnamic acid matrix.

### 2.4. Aerosol Droplet Size Analysis

Aerosol droplet size was initially characterised using laser diffraction as previously described [26]. Briefly, the devices were loaded with 0.5 mL of the sample and connected to the inlet of the droplet sizer (Spraytec, Malvern, UK). The nebuliser under test was turned on and run until the entire dose was delivered. Testing was carried out in triplicate.

In a separate series of experiments, cascade impaction (NGI, Copley Scientific, Nottingham, UK) was used to assess the aerodynamic diameter of the nebuliser-generated aerosol. The nebuliser (Aerogen Solo) was attached to the apparatus via a plastic connector and metal inlet (throat). The vacuum pump flow was 15 L/min with either 1 or 2 mL of a 1 mg/mL solution prepared in distilled water. After nebulisation was complete, the apparatus was dismantled and the eight plates, connector, throat, nebuliser, and capture filter (Respigard 303, Baxter, Dublin, Ireland) were all thoroughly rinsed with distilled water to collect the impacted peptide. The washings of each stage were analysed by HPLC and compared to a standard line of peptide concentration versus peak area. The % deposition in each stage was used to calculate the mass median aerodynamic diameter (MMAD) of the spray and the mass balance of each stage. This was carried out three times for each peptide. Samples of low concentration were lyophilised and reconstituted in one-tenth the original volume to increase the HPLC signal and improve accuracy.

### 2.5. Breathing Apparatus

The Aerogen Solo was used to nebulise 2 mL of a 1 mg/mL solution in distilled water into a breathing apparatus (Ingmar Medical, Pittsburgh, PA, USA). The conditions of a normal human breathing pattern were simulated using a Salter valved facemask 81070-0 (Salter, El Paso, TX, USA) and an ASL 5000 active servo lung (IngMar Medical, Pittsburgh, PA, USA). The parameters were 15 breaths/min, an inhalation:expiration ratio of 1:1, a tidal volume of 500 mL, and a 2 L of supplementary oxygen flow which were mechanically ventilated. The nebulised peptides were collected distal to the model on a capture filter which was washed afterwards with 10 mL of distilled water. The nebuliser was also washed. The washings were analysed by HPLC and the % of the original dose delivered to the capture filter was calculated, giving the % delivered to the lung. This was carried out three times for each peptide.

### 2.6. Bacterial Susceptibility Testing

Minimum Inhibitory Concentrations (MICs) were determined as described previously using the broth microdilution method according to the guidelines of the Clinical and Laboratory Standards Institute (CLSI) [27], with modifications for cationic peptides as described by Wu and Hancock [28]. Briefly, serial doubling dilutions of peptides were prepared at 10 times the final assay concentration (2.5 µg/mL–640 mg/mL) in a sterile solution containing 0.2% *w*/*v* bovine serum albumin (BSA) and 0.01% *v*/*v* acetic acid. Assays were prepared in 96-well plates where triplicate wells contained 10 µL of each 10× peptide dilution and approximately 1.4 × 10^4^ CFU/well of bacterial inoculum in 100 µL Mueller–Hinton (MH) broth (non-cation adjusted, Oxoid, UK) resulting in final peptide concentrations of 0.25–64 µg/mL. The MIC was recorded as the lowest peptide concentration showing no visible growth after approximately 18 h incubation with reference to positive growth control (no peptide) and negative controls (no inoculum).

## 3. Results

### 3.1. Characterisation of Pro- and AAG-WMR after Nebulisation

Aqueous solutions of 1 mg/mL of pro-WMR and AAG-WMR were both nebulised using an Aerogen Solo. Both peptides were analysed before and after nebulisation using electrospray mass spectrometry and HPLC (Figures S1 and S2). They had comparable mass spectra and chromatograms before and after nebulisation with no additional peaks showing (see supplementary figures for further information). A comparison of the MICs of the inactive pro-WMR and active AAG-WMR before and after nebulisation was also made. No difference in MICs was observed against *P. aeruginosa* strain PAO1 and three CF clinical isolates (Table 1).

### 3.2. Aerodynamic Size Distribution of the Peptides

Analysis of the aerosol droplet size of a 1 mg/mL aqueous solution by laser diffraction gave comparable volume mean diameters (VMD) for both peptides: 3.79 μm and 3.80 μm for pro- and AAG-WMR, respectively, with accompanying geometric standard deviations (GSD) of 1.76 and 1.74. The proportion of droplets with a diameter below 5 μm, i.e., the fine-particle fraction (FPF) was above 65% for both peptides. The results of the cascade impaction were in general agreement with the laser diffraction. The MMADs were 3.59 μm and 3.14 μm for pro- and AAG-WMR, respectively, using 1 mL of solution. These studies were repeated using 2 mL of the solution using different batches of the peptide (in order to see if the increasing volume or different batches affected the MMAD) and the results were in close agreement with the 1 mL results, 3.92 μm and 3.80 μm for pro- and AAG-WMR respectively (Table 2).

**Figure 1:**
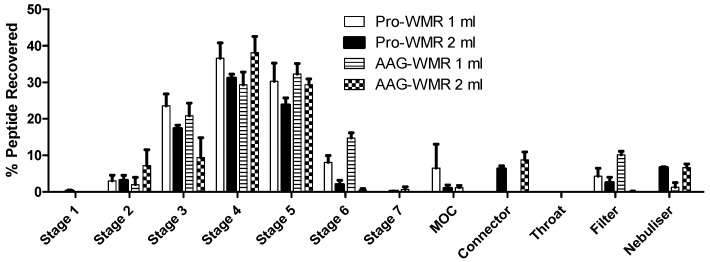
The mean ± SEM of % peptide recovered from each stage of the next generation impactor for pro- and AAG-WMR, *n* = 3. Included are stages 1 to 7, the micro-orifice collector (MOC), the connector, throat, and filter stages, as well as the remaining peptide in the nebuliser. Each increasing stage number of the impactor has a smaller cut-off diameter, with MOC being the smallest. After nebulisation of 1 or 2 mL of 1 mg/mL peptide solution, each stage was washed with dH_2_O and the washings analysed by HPLC to determine the drug deposition. The figures were used to calculate MMAD.

The fractions recovered from the different stages of the impactor indicated that the majority of both peptides settled in Stages 4 and 5, which had a cut-off diameter of 3.3 μm and 2.08 μm, respectively (Figure 1).

### 3.3. Aerosol Delivery during Simulated Breathing

**Figure 2:**
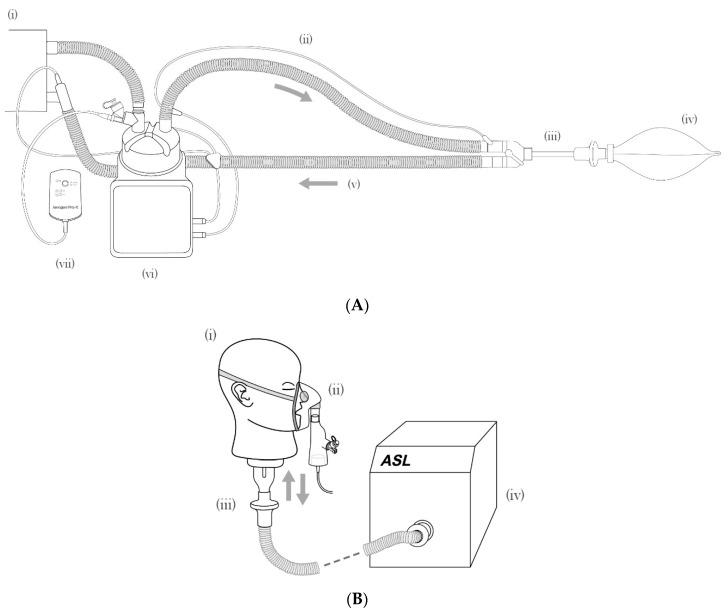
(**A**) The mechanical ventilation: (i) mechanical ventilator, (ii) inspiratory limb, (iii) endotracheal tube and collection filter, (iv) lung, (v) expiratory limb, (vi) humidifier, (vii) nebuliser controller and nebuliser. (**B**) Spontaneous breathing: (i) Adult head model, (ii) Aerogen Solo and Ultra with facemask, (iii) collection filter, (iv) breathing simulator. Note: flow direction indicated by arrows.

**Figure 3:**
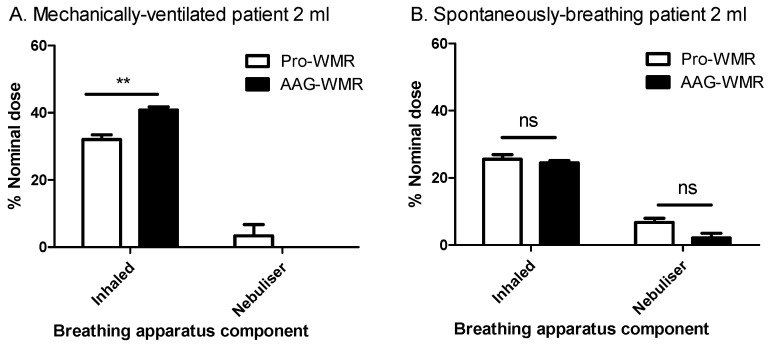
The mean ± SEM of % peptide input for each stage of a breathing apparatus for pro- and AAG-WMR, showing (**A**) the % peptide recovered from the inhalation capture filter and the nebuliser of 2 mL of 1 mg/mL peptide solution from a spontaneously-breathing patient, *n* = 3; (**B**) the % peptide recovered from the inhalation capture filter and the nebuliser of 2 mL of 1 mg/mL peptide solution for a mechanically ventilated patient, *n* = 3. ** denotes *P* < 0.01, ns denotes *P* > 0.05.

In a model of a mechanically-ventilated patient (Figure 2A), the quantity of peptide collected on the inhalation capture filter (representing the level of the bifurcation of the lung) was compared to the nominal dose. This allowed the percentage delivery to be calculated. Deliveries of 32.1 ± 1.4% and 40.8 ± 0.9% were observed for pro-WMR and AAG-WMR respectively, with the difference being statistically significant (*P* = 0.0067, Figure 3A).

Lower recovery levels were noted using a different patient model, that of a spontaneously-breathing healthy adult (Figure 2B). For pro-WMR, 25.6 ± 1.4% of the initial dose was delivered to the lung, while 24.4 ± 0.7% of AAG-WMR was delivered. The difference between both peptides was not statistically significant (*P* > 0.05, Figure 3B).

In all cases, it can be seen that very little peptide remained in the nebuliser, with the highest figure for a single run being 9.2% and below 5% in most cases. The time taken to nebulise 1 mL was comparable for both peptides, being approximately 170 s.

## 4. Discussion

This study demonstrates that it is possible to generate a respirable aerosol of a pro-AMP. This prodrug, but also its active peptide component were both unchanged after vibrating mesh nebulisation and maintained their levels of antimicrobial activity against the most common CF pathogen, *P. aeruginosa*. This is particularly important for pro-WMR, as the integrity of the toxicity-lowering modification was maintained (evident from both mass spectrometry, HPLC, and MIC experiments). Additionally, these results indicate that the net charge reduction (+6 in AAG-WMR to +1 in pro-WMR) caused by the oligo-glutamic acid pro-moiety, which could potentially result in the aggregation of the prodrug sequence by relieving its electrostatic repulsion, does not seem to significantly impact on the physical characteristics of this peptide in the context of its delivery to the lung. As discussed earlier, loss of activity, aggregation and/or degradation after nebulisation have been observed with other biomolecules such lactate dehydrogenase [14,17], IgG1 and the autoimmune treatment receptor SM101 [17], and naked siRNA [29] in combination with other aerosol generator technologies.

The current formulation of a simple aqueous solution could feasibly be used clinically with little modification, perhaps only pH-adjustment and filter sterilisation. The fact that the potentially toxic effects of the active peptide are already neutralised by the pro-moiety [5] can be considered a major advantage as there is no requirement for additional modifications to the nebulisation formulations. Previously, the antimicrobial peptide CM3 was encapsulated in liposomes for lung delivery due to issues with toxicity and stability, a strategy that can be successful [30] but complicates the formulation and has associated risks such as liposomal leakage during nebulisation, which has been observed with SLPI [31]. Liposomal encapsulation can also reduce the antimicrobial activity of nebulised antibiotics, as has been observed with the peptide polymyxin B [32].

The physical characteristics of the generated aerosol have a major impact on how it is deposited in the airways and considering that infection is localised to the lower airways, affects antimicrobial action. The MMAD of both peptides was in the range of 1-5 μm required for lower airway deposition [11], and the additional pro-moiety of pro-WMR does not appear to significantly affect the droplet size. Pro-WMR is targeted for cleavage and activation by NE in the endobronchial space in order to limit the toxicity of the active sequence. The combination of this targeting with controlled aerosol droplet size may serve to allow peptides that were formerly considered too toxic to be used to treat infection in CF. Parallels could be drawn in future with the peptide colistin, now delivered via inhalation in CF as the prodrug colistimethate sodium [6], which was also originally not used for reasons of toxicity, reconsidered with the increasing emergence of antibiotic resistance [33].

The results from the simulated breathing patient experiments demonstrate that a large proportion of the nominal peptide dose can be expected to reach the lung and compares well to established therapeutics. For comparison with the current in vivo efficiencies, in a group of male CF patients receiving 150 mg of tobramycin from a Pari^®^ eFlow VMN, 28.3% was deposited in the lung [34]. While a larger percentage of the AAG-WMR was delivered in the mechanically ventilated model, the difference between pro- and AAG-WMR was not profound and was not replicated in the spontaneously-breathing model. It is demonstrated that the additional amino acids and the net charge modification of the pro-drug strategy are not detrimental to pulmonary delivery. Higher delivery efficiencies were observed in the mechanically-ventilated model compared to the spontaneously-breathing model (40.8 ± 0.9% vs. 24.4 ± 0.7%, respectively, for AAG-WMR). This may be attributed to factors such as the nebuliser position and bias flow influencing aerosol delivery during mechanical ventilation [35,36]. Even greater delivered doses could potentially be achieved through the use of breath-triggered aerosol generation whereby aerosol is only generated and delivered to the patient during the inspiratory phase of the breath [37,38].

The relatively short treatment time (approximately 170 s per dose) also compares well to that previously observed with tobramycin delivery using a Pari^®^ eFlow VMN [32]. The potential clinical delivery times per dose will depend on the desired delivered dose to the lungs and the concentration of the peptide solution. This is an important practical consideration when the high treatment burden of CF patients is taken into account. Additionally, the low level of residual peptide remaining in the nebuliser and the model lung experiments (below 5% in nearly all cases) confirms the utility of VMNs for delivering expensive peptides.

In these experiments, nebulised amounts of 1 and 2 mg were investigated, which will likely be insufficient for clinical use. This is made more difficult by relatively high MICs of the active peptide (8–32 μg/mL or 4.3–17.4 μM) against *P. aeruginosa*, coupled with the fact that the MIC may need to be greatly exceeded locally to overcome inhibitory effects of the CF airway surface liquid and mucus. However, previously it was seen that the active sequence (which is mostly composed of protease-resistant D-amino acids) can still exert its antimicrobial effects in CF bronchoalveolar lavage fluid [5]. It is not possible to accurately predict what amount of peptide would need to be nebulised for effective the in vivo antimicrobial effect and, thus, relate the results here to a potential clinical dose. Sputum levels of tobramycin after a single dose of 300 mg/5 mL in one study, though highly variable, were found to greatly exceed the reported MICs against *P. aeruginosa* (0.5–32 μg/mL or 1.1–68.4 μM). However, high sputum levels could also be indicative of high upper airway deposition [19]. A mathematical model was applied to predict the lung deposition of liposomal polymyxin B. The nebulisation of 25 mg was expected to provide a concentration in excess of the MIC (62.5 μg/mL or 48 μM) for a large part of the conducting airways, although this was affected by parameters such as mucus production and velocity [32].

Determining the therapeutic dose was not the aim of this study, rather, it was to investigate the feasibility of nebulising a pro-AMP for CF infection, which is demonstrated. Future work may investigate higher volumes and concentrations of pro-AMP solutions (and perhaps different pro-AMP sequences). Respirable aerosols were generated for both 1 and 2 mL of solution and, when compared to current treatment regimens, it seems that increasing the concentration may bridge the gap for clinical studies. While the increased amounts of peptide required may represent an economic hindrance, there are already high costs associated with inhaled antibiotic therapy in CF which can be offset against the reduction in expensive hospital stays; economic evaluations have shown the benefit of inhaled tobramycin therapy in this regard [6].

## 5. Conclusions

Following on from previous studies into antimicrobial activity and host cytotoxicity, this study provides further support for the development of pro-AMPs as therapeutics in CF. It is possible that pro-WMR may not represent the final optimised pro-AMP sequence, however, the investigation into its nebulisation here demonstrates that the pro-AMP modification is not detrimental to lung delivery and should be pursued further for treating pulmonary infection in CF.

## Figures and Tables

**Table 1:** The Minimum Inhibitory Concentrations (MIC) values for pre- and post-nebulisation HDPs vs. *P. aeruginosa* PAO1 and clinical isolates (PABH01-03).

Peptide	MIC vs. *P. aeruginosa* Strains (μg/mL)			
	PAO1	PABH01	PABH02	PABH03
AAG-WMR	32	8	16	32
AAG-WMR (nebulised)	32	8	16	32
Pro-WMR	>64	>64	>64	>64
Pro-WMR (nebulised)	>64	>64	>64	>64

**Table 2:** The fine particle fraction under 5 μm, mean mass median aerodynamic diameters (with 1 and 2 mL delivery) and volumetric mean diameters (with associated geometric standard deviations) of pro- and active antimicrobial peptides (AMPs), determined using both laser diffraction and cascade impaction *n* = 3.

	Pro-WMR	AAG-WMR
MMAD (GSD) 1 mL	3.59 μm (1.67)	3.14 μm (1.93)
MMAD (GSD) 2 mL	3.92 μm (1.61)	3.86 μm (1.51)
VMD (GSD)	3.79 μm (1.76)	3.80 μm (1.74)
FPF < 5 μm	67.1%	66.6%

## Supplementary Materials

The following are available online at https://www.mdpi.com/1999-4923/11/5/239/s1, Figure S1: HPLC and electrospray ionisation mass spectrometry of AAG-WMR before and after nebulisation, Figure S2: HPLC and electrospray ionisation mass spectrometry of pro-WMR before and after nebulisation.
