# Supplementary material for: Vibrating Mesh Nebulisation of Pro-Antimicrobial Peptides for Use in Cystic Fibrosis
Source: Pharmaceutics. 2019 May 17;11(5):239. doi: 10.3390/pharmaceutics11050239 (PMC6571777; doi:10.3390/pharmaceutics11050239)
Supplement: Supplementary file 1 [file pharmaceutics-11-00239-s001.pdf]

pharmaceutics

Supplementary Materials: Vibrating Mesh Nebulisation of Pro-Antimicrobial Peptides for Use in Cystic Fibrosis

Éanna Forde, Graeme Kelly, Louise Sweeney, Deirdre Fitzgerald-Hughes, Ronan MacLoughlin and Marc Devocelle

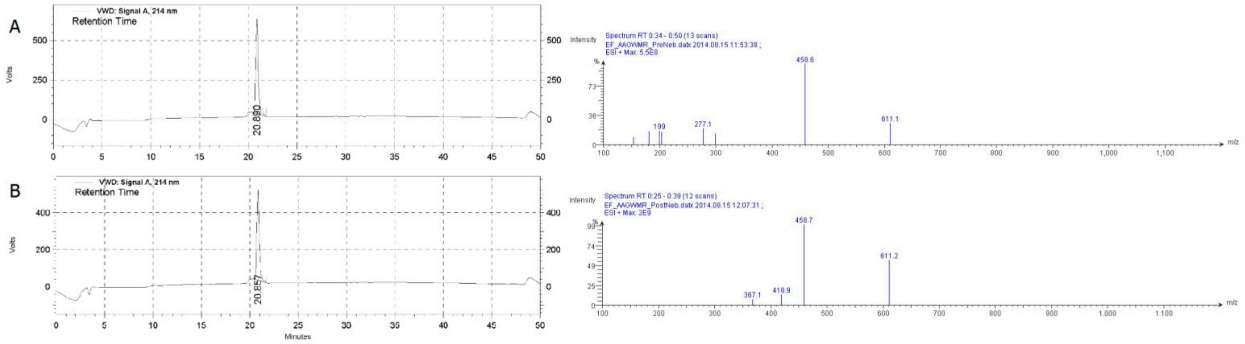

Figure S1. HPLC and electrospray ionisation mass spectrometry of AAG-WMR before (**A**) and after (**B**) nebulisation. Retention times 20.890 min and 20.857 min. The signals at *m*/*z* = 611.1, 458.6, and 367.1 are the [M +3 H]$^{3+}$, [M +4 H]$^{4+}$, and [M +5 H]$^{5+}$respectively for AAG-WMR.

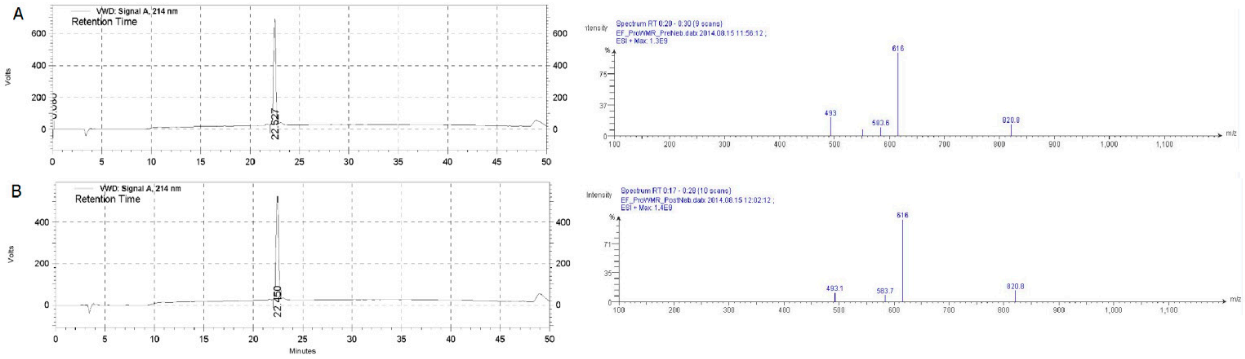

Figure S2. HPLC and electrospray ionisation mass spectrometry of pro-WMR before (**A**) and after (**B**) nebulisation. Retention times 22.527 min and 22.450 min. The signals at *m*/*z* = 820.8, 616, and 493 are the [M +3 H]$^{3+}$, [M +4 H]$^{4+}$, and [M +5 H]$^{5+}$respectively for pro-WMR.

© 2019 by the authors. Submitted for possible open access publication under the terms and conditions of the Creative Commons Attribution (CC BY) license (http://creativecommons.org/licenses/by/4.0/).
